# The use of a UV-C disinfection robot in the routine cleaning process: a field study in an Academic hospital

**DOI:** 10.1186/s13756-021-00945-4

**Published:** 2021-05-29

**Authors:** Füszl Astrid, Zatorska Beata, Van den Nest Miriam, Ebner Julia, Presterl Elisabeth, Diab-Elschahawi Magda

**Affiliations:** Department of Infection Control and Hospital Epidemiology, Medical University Vienna, Währinger Gürtel 18-20, 1090 Vienna, Austria

**Keywords:** Healthcare-associated infections, Infection control, Ultraviolet-C, UV-C robot, *Candida auris*

## Abstract

**Background:**

Environmental surface decontamination is a crucial tool to prevent the spread of infections in hospitals. However, manual cleaning and disinfection may be insufficient to eliminate pathogens from contaminated surfaces. Ultraviolet-C (UV-C) irradiation deploying autonomous disinfection devices, i.e. robots, are increasingly advertised to complement standard decontamination procedures with concurrent reduction of time and workload. Although the principle of UV-C based disinfection is proven, little is known about the operational details of UV-C disinfection delivered by robots. To explore the impact of a UV-C disinfection robot in the clinical setting, we investigated its usability and the effectiveness as an add-on to standard environmental cleaning and disinfection. Additionally, its effect on *Candida auris*, a yeast pathogen resistant to antifungals and disinfectants, was studied.

**Methods:**

After setting the parameters “surface distance” and “exposure time” for each area as given by the manufacturer, the robot moved autonomously and emitted UV-C irradiation in the waiting areas of two hospital outpatient clinics after routine cleaning and/or disinfection. To quantify the efficacy of the robotic UV-C disinfection, we obtained cultures from defined sampling sites in these areas at baseline, after manual cleaning/disinfection and after the use of the robot. Four different *C. auris* strains at two concentrations and either in a lag or in a stationary growth phase were placed in these areas and exposed to UV-C disinfection as well.

**Results:**

The UV-C irradiation significantly reduced the microbial growth on the surfaces after manual cleaning and disinfection. *C. auris* growth in the lag phase was inhibited by the UV-C irradiation but not in the presence of the rim shadows. The effects on *C. auris* in the stationary phase were differential, but overall *C. auris* strains were not effectively killed by the standard UV-C disinfection cycle. Regarding usability, the robot’s interface was not intuitive, requiring advanced technical knowledge or intensive training prior to its use. Additionally, the robot required interventions by the technical operator during the disinfection process, e.g. stopping due to unforeseen minor dislocation of items during the clinical service or due to moving individuals, making it a delicate high-tech device but not yet ready for the autonomous use in the clinical routine.

**Conclusions:**

Presently, the UV-C robot tested in this study is not ready to be integrated in the environmental cleaning and disinfection procedures in our hospital. The single standard disinfection UV-C irradiation cycle is not sufficient to inactivate pathogens with augmented environmental resilience, e.g. *C. auris*, particularly when microbial loads are high.

**Supplementary Information:**

The online version contains supplementary material available at 10.1186/s13756-021-00945-4.

## Introduction

Healthcare-associated infections (HAIs) are a major complication of medical treatment and care, necessitating a prolonged hospital stay and causing morbidity associated with increased costs and last but not least increased mortality [1]. Up to 7% of the patients in developed and 10% of the patients in developing countries are at risk to acquire at least one HAI, most of which may be prevented through infection prevention and control (IPC) measures [2].

Pathogens, e.g. methicillin-resistant *Staphylococcus aureus* (MRSA), vancomycin-resistant Enterococcus (VRE), *Clostridium difficile,* Norovirus and fungi are viable on surfaces for prolonged periods [3–6]. As a result, environmental contamination leads to an increased risk of HAIs [3, 7]. To prevent HAIs and the spread of pathogens via contaminated surfaces, hospital rooms have to be cleaned and disinfected at regular intervals by trained personnel. For decontamination in hospitals, cleaning agents and disinfectants approved by technical expert committees must be used. However, manual cleaning and disinfection is time and personnel consuming and—due to lack of time and training—sometimes not sufficient. Erratic cleaning and disinfection processes, wrong choice of the appropriate formulation of cleansers or disinfectants and non-adherence to the required contact time of disinfectants may impair the efficacy of standard approaches. Studies have shown that more than 50% of surfaces may go untouched by manual cleaning [3, 8, 9]. Secondly, in times of crisis, the supply of disinfectants may be disrupted, as has been demonstrated in the current COVID-19 pandemic [10].

Because of the shortcomings of routine environmental decontamination as mentioned above, autonomous touchless surface disinfection technologies have evolved. By disrupting the structure of DNA or RNA of microorganisms, UV-C irradiation at a wavelength of 254 nm is most effective in killing bacteria, viruses, fungi, and even spores (in falling order of effectiveness) [11].

Previous studies indicate that disinfection technologies using UV-C irradiation are an enhancement to standard cleaning and disinfection, reducing the environmental microbial burden and potentially mitigating the risk of acquiring a HAI [12–18]. This has been demonstrated for different pathogens such as MRSA, *Clostridium difficile* and VRE [13, 18] and in different clinical settings such as ambulances [19], inpatient rooms [16, 20] and operating theaters [15].

The efficacy of UV-C irradiation to inactivate microorganisms depends on a number of factors including varying resistance levels of different microorganisms to UV-C light, the initial inoculum and the UV-C dose received, which is a result of distance, duration of exposure and the presence of shadows [21]. Organic soils, furniture, draperies or other healthcare equipment etc. are the most common cause of shadows. Shadows drastically reduce the efficacy of UV-C irradiation. To remove soils, surfaces must be cleaned manually before applying UV-C irradiation. UV-C efficacy also declines with increasing distance of the UV-C source to the surfaces.

*Candida auris* is an emerging, multidrug-resistant yeast pathogen first described in 2009 as the cause of multiple nosocomial outbreaks worldwide, leading to severe infections and high mortality rates [22]. *C. auris* poses a particular challenge for IPC in hospitals because it can stay viable on surfaces for prolonged periods and is resistant to several commonly used disinfectants [25]. Consequently, the hospital environment is considered an important reservoir for transmission [22–25]. Further, compared to other pathogens, *C. auris* is resistant to UV-C light and needs extended exposure to UV-C irradiation to induce growth inhibition [26, 27].

UV-C disinfection robots have been increasingly employed in different settings such as hospitals, airports and shopping malls as a result of the COVID-19 pandemic [28]. However, little information is available on their efficacy and usability in the routine cleaning and disinfection process in hospital settings. To shed some further light on operational aspects, we aimed to test a new UV-C robot in real life. To evaluate the antimicrobial efficacy of a standard UV-C disinfection cycle, we investigated its effect on the microbial burden on clinical surfaces when applied after standard terminal cleaning and disinfection (STC&D) in the waiting areas of two outpatient clinics. As a surrogate for resilient microorganisms, four different *C. auris* strains in varying densities (10^3^ and 10^6^ CFUs/ml) and different growth characteristics (lag vs stationary growth phase) were placed within these areas and exposed to UV-C irradiation as well.

## Materials and methods

### UV-C light emitting disinfection device

**Fig. 1:**
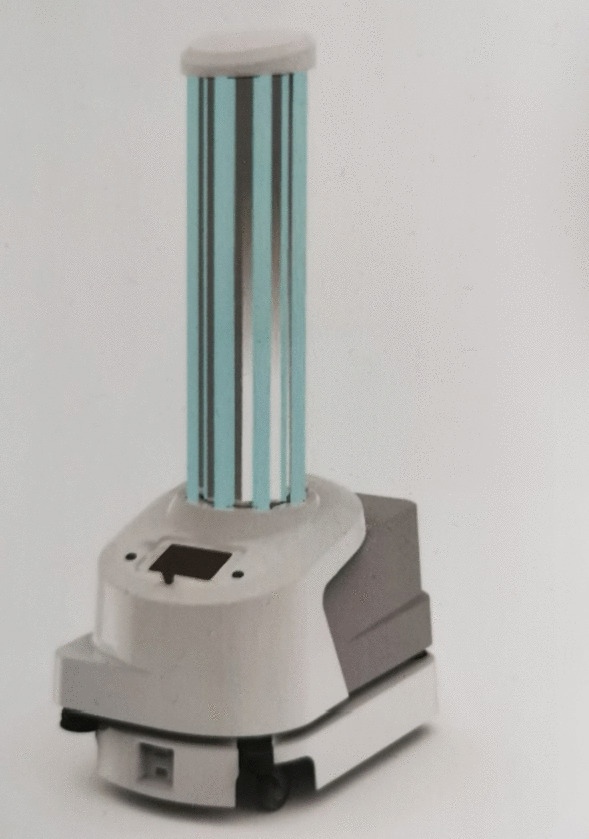
UVD Robot® (Clean Room Solutions)

We studied the self-driving Ultra Violet Disinfection Robot® (UVD-R) by Clean Room Solutions because it was the most advanced UV-C irradiation device available for autonomous use (Fig. 1).

This robot moves autonomously in a pre-defined area after being programmed for the parameters exposure time and distance of surfaces. It consists of eight lamps that are located on top of a platform. During a disinfection cycle, they emit UV-C irradiation at a wavelength of 254 nm, enabling a 360 degree coverage. During the disinfection process, the UV-C light emitting robot moves at 10 cm per second, providing a dose of 2.7 mJ/cm^2^ per second for directly exposed surfaces in 1 m distance and achieving a coverage of areas at a distance of several meters (according to manufacturer's specifications). However, it is worth bearing in mind that the UV-C light intensity over distance is governed by the inverse square law, resulting in significantly smaller doses for areas further away from the device. To enable autonomous moving, the robot must be pre-programmed using a detailed map of the position of furniture and other obstacles in the area to be treated with UV-C irradiation. Once every parameter is set, furniture and all other objects must remain in exactly the same place to enable an autonomous functioning. Due to the high-intensity UV-C irradiation, the UV-C robot may only be used in rooms devoid of people. Unintentional exposure leads to cutaneous erythema and photokeratitis. For safety, this UV-C robot automatically shuts off when its motion sensor detects any moving individuals during the disinfection process.

### Setting

Between July 23rd and August 2nd 2020, the study was performed in the waiting areas of two outpatient clinics (ear, nose and throat medicine and oncology waiting areas with a size of 137 m^2^ each) of Vienna General Hospital (VGH), a 1728 bed tertiary hospital in the capital of Austria. During the study period, 347 patients were treated in the ENT (23/07–26/07) and 400 patients in the oncology outpatient department (29/07–02/08).

In one of the outpatient areas, manual cleaning/disinfection was carried out by in-house cleaning personnel while the other outpatient clinic was served by a cleaning service providing company. Cleaning and disinfection followed the standard operating procedures (SOP) of VGH: Floors in the outpatient waiting areas are manually cleaned once a day while chairs and tables are disinfected once a day using either alcohol-based products or products based on active oxygen (*Descogen*® *Liquid*)*.*

**Fig. 2:**
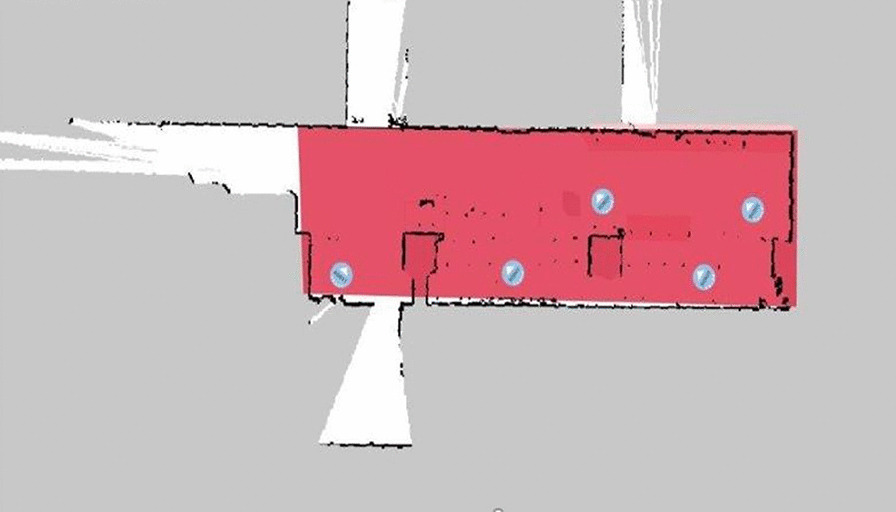
Area map pre-programmed into the UV-C robot in the ENT outpatient clinic. The blue dots indicate where the robot had to stop for 3 min during the disinfection cycle. The red color indicates which area was covered by the mapping procedure and exposed to UV-C light

**Fig. 3:**
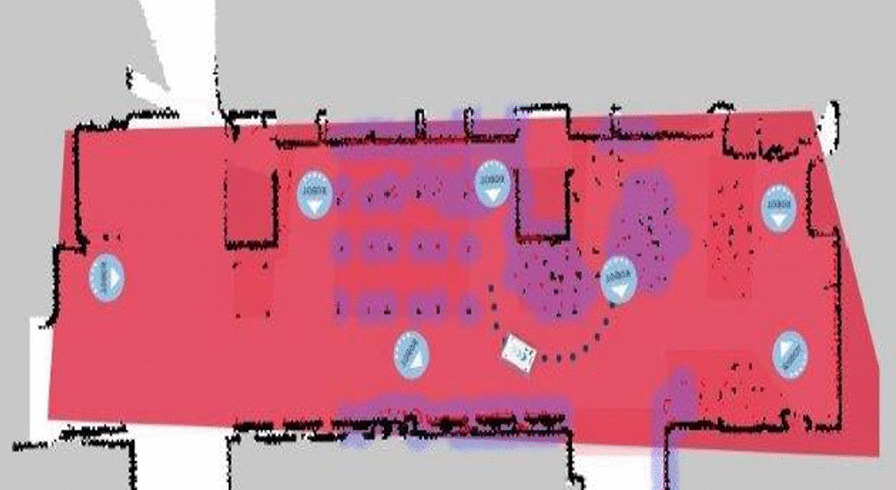
Area map pre-programmed into the UV-C robot in the oncology outpatient clinic. The blue dots indicate where the robot had to stop for 3 min during the disinfection cycle. The red color indicates which area was covered by the mapping procedure and exposed to UV-C light. The violet color indicates “light detection and ranging” (Lidar), which is a way for the robot to see an obstacle and avoid that area

Prior to the start of the study, a member of the UVD Robot® installation team pre-programmed area maps with the exact position of furniture and other items to enable autonomous disinfection cycles. For the pre-programming, the team inspected the two outpatient clinics to map the robot’s route and identify critical areas that required longer UV-C light exposure. The robot was pre-programmed to stop at various predefined positions for 3 min to achieve optimal UV-C exposure of all relevant surfaces (Figs. 2, 3).

The device was used after the room had been manually cleaned and disinfected according to SOP. The procedure was then initiated remotely once all doors had been closed. Each disinfection cycle was completed within 20–25 min per outpatient setting.

### Sampling procedure

To evaluate the robot’s effect on residual contamination, samples were collected from different surface sites before and after routine cleaning and/or disinfection, and after the use of the UV-C robot. Surface sites selected for sampling included high-touch surfaces and remote sites supposedly to be out of reach for easy cleaning and those that appeared unlikely to achieve full exposure to UV-C irradiation.

In the ENT waiting area, six sites were sampled (wall, armrests of two different chairs, back of a chair, wooden play element for children, window countertop) (see Additional file 1: Table S1).

In the oncology waiting area, sampling was performed from eight different sites (patient registration area, table surface next to patient registration, armrests of two different chairs, window countertop, push button of a vending machine, leaflet dispenser) (see Additional file 1: Table S2).

**Fig. 4:**
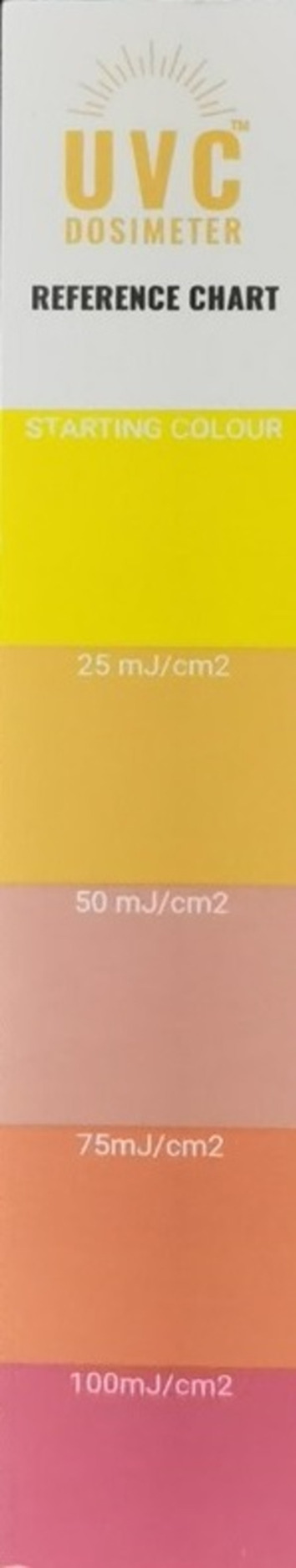
Reference chart (UV-C dose received according to indicator’s change of color); Intellego Technologies

To monitor the amount of exposure to UV-C irradiation, disposable indicators were placed on all surfaces used for sampling before initiating the UV-C cycle. The indicators changed color depending on the achieved dose, corresponding to doses ranging from 25 mJ/cm^2^ in shadowed areas to 100 mJ/cm^2^ at the most highly exposed sites (Fig. 4).

The achieved UV-C doses corresponding to each sampling site are presented in the supplemental material (see Additional file 1: Tables S1–S2).

### Microbiological methods

#### Determination of the microbial burden on hospital surfaces

We collected environmental contact cultures from each sampling site using Tryptic Soy Agar (TSA) plates with a diameter of 5.5 cm (VWR International, Vienna, Austria). Samples were collected on 9 days by the same lab technician following a predefined standardized sampling scheme:

During the study period, sampling was performed three times per study day:

- before routine cleaning and/or disinfection,
- after routine cleaning and/or disinfection, and
- after the use of the UV-C robot.

After sampling, TSA plates were incubated at 37 °C for 48 h. Following incubation, the number of colony forming units (CFUs) on each plate was counted. Subsequently, the colonies were identified using the MALDI-TOF mass spectrometry method (Bruker, USA).

Surfaces were subdivided into three categories according to their level of contamination used routinely for environmental samples at our institution:

1. surfaces with a low microbial burden, defined as 0–3 CFUs/24 cm^2^
2. surfaces with an average microbial burden, defined as 4–50 CFUs/24 cm^2^
3. surfaces with a high microbial burden, defined as > 50 CFUs/24 cm^2^.

#### Preparation of *Candida auris* strains

We investigated whether the type of *C. auris* strain, seeding density and incubation prior to UV-C light exposure affected UV-C efficacy.

To study potential differences in sensitivity to UV-C irradiation, four *C. auris* strains were evaluated: *C. auris* NCPF 8971, *C. auris* NCPF 8977, *C. auris* NCPF 8984 and *C. auris* DSM 21092.

Plates containing Sabouraud Dextrose Agar (SDA) (Becton Dickinson, Franklin Lakes, USA) were inoculated with 100 µl of *C. auris* suspension at two different concentrations, 10^3^ CFUs/ml and 10^6^ CFUs/ml respectively.

Each strain of *C. auris* suspension containing 10^3^ CFUs/ml was spread on three SDA plates and incubated for 24 h at 30 °C. For the field experiment, rimless TSA plates were pressed on these SDA plates, mimicking surface contamination by hands and fomites as demonstrated by Adams et al. [29]. Overall, 12 TSA plates were used per study day.

Additionally, each strain of *C. auris* suspension containing 10^6^ CFUs/ml was spread on one SDA plate without further incubation, yielding four plates with *C. auris* in a lag phase per study day. Then, *C. auris* exposed TSA plates (10^3^ CFUs/ml, incubated overnight) as well as inoculated SDA plates (10^6^ CFUs/ml without further incubation) were placed on two tables in the waiting area of the oncology outpatient clinic during the standard disinfection cycle. Indicators were placed alongside that measured the UV-C dose received. This experiment was performed in triplicate.

Following UV-C exposure, all plates were incubated at 30 °C for 7 days. Then, *C. auris* growth was compared to unexposed controls.

### Statistical analysis

Standard descriptive analysis was done to summarize the microbiological findings. Differences between the number of CFUs after standard terminal cleaning and disinfection compared to the combined use of STC&D and UV-C irradiation were analyzed using the Wilcoxon matched-pairs signed rank test. Statistical analysis was performed at a two-sided significance level of 0.05, using SPSS (Version 26.0, IBM).

In an exploratory data analysis, the plates of UV-C exposed *C. auris* strains with a concentration of 10^6^ CFUs/ml were visually compared to unexposed controls. Results of *C. auris* with an initial concentration of 10^3^ CFUs/ml were quantified as the number of CFUs, comparing exposed and unexposed plates.

## Results

### Effects on the environmental microbial burden

During the study period, we collected 192 samples (72 in the ENT and 120 in the oncology outpatient areas, respectively) from 14 sites (64 samples prior to any cleaning and disinfection, 64 after manual cleaning and disinfection and 64 after the use of the UV-C robot). Prior to manual cleaning, the surfaces most heavily contaminated were the armrests of chairs, followed by the window countertops. The least contaminated sites were the walls, the leaflet dispenser and the backs of patient chairs. The leaflet dispenser, however, was empty during the study period according to an in-house IPC order to avoid potential cross-transmission via contaminated leaflets during the COVID-19 pandemic. In general, contamination levels prior to any cleaning and disinfection were higher in the oncology outpatient area than in the ENT outpatient area.

In Table 1, the level of contamination according to the time of sampling is summarized for each outpatient waiting area. UV-C indicators showed that some of the sites received a suboptimal UV-C dose. Nonetheless, the additional use of UV-C irradiation achieved a further reduction of CFUs compared to standard cleaning and/or disinfection, resulting in decontamination of 96.9% (62/64) of the surfaces compared to decontamination of 50.0% (32/64) of the surfaces after manual cleaning and disinfection alone.

**Table 1:** Proportion of contact cultures with low, acceptable and high microbial burden before routine cleaning and/or disinfection, after routine cleaning and/or disinfection and after the use of the UV-C robot

	ENT outpatient area			Oncology outpatient area		
	Low	Average	High	Low	Average	High
Before C&D	45.8% (11/24)	37.5% (9/24)	16.7% (4/24)	22.5% (9/40)	60.0% (24/40)	17.5% (7/40)
After C&D	79.2% (19/24)	20.8% (5/24)	0% (0/24)	32.5% (13/40)	62.5% (25/40)	5.0% (2/40)
After C&D + UV-C	100% (24/24)	0% (0/24)	0% (0/24)	95.0% (38/40)	5.0% (2/40)	0% (0/40)

*C&D* cleaning and disinfection, *UV-C* ultraviolet C; low = 0–3 CFUs/24 cm^2^, average = 4–50 CFUs/24 cm^2^, high > 50 CFUs/24 cm^2^

With regard to the microbial burden, the additional use of the UV-C robot significantly decreased the median number of CFUs in both outpatient areas compared to manual cleaning and disinfection alone (*p* = 0.008 and *p* < 0.001, for the ENT and for the oncology outpatient areas, respectively) (Table 2).

**Table 2:** Reductions in Colony-Forming Units after routine cleaning and/or disinfection compared to baseline and after routine cleaning and/or disinfection + UV-C irradiation compared to routine cleaning and/or disinfection alone

	ENT outpatient area					Oncology outpatient area				
	No. of samples	Median CFU (IQR)	Min	Max	*p* value	No. of samples	Median CFU (IQR)	Min	Max	*p* value
Before C&D	24	8.5 (8.5–28.3)	0	207	0.003	40	22.0 (4.3–36.0)	0	200	
After C&D	24	0 (0–2.8)	0	18		40	6.5 (2.3–20.5)	0	101	<0.001
After C&D	24	0 (0–2.8)	0	18	0.008	40	6.5 (2.3–20.5)	0	101	
After C&D + UV-C	24	0 (0–0)	0	1		40	0 (0–0)	0	5	<0.001

*No.* number, *CFU* Colony Forming Unit, *IQR* interquartile range, *Min* minimum, *Max* maximum, *C&D* cleaning and disinfection, *UV-C* ultraviolet C

### Qualitative description of the environmental microbiome

Most bacterial isolates were classified as physiological skin flora (222/297; 74.7%). Next, 13.1% of bacteria were classified as environmental microorganisms (39/297), 6.4% of bacteria were classified as oropharyngeal flora (19/297) and 5.7% of bacteria as potential pathogens (17/297).

Typical pathogens were *Staphylococcus saprophyticus* (n = 5) and *Staphylococcus lugdunensis* (n = 1), *Acintetobacter baumanii* (n = 2), *Aerococcus viridans* (n = 1), *Streptococcus pneumonia* (n = 1), *Staphylococcus aureus* (n = 1) *and Enterococcus casseliflavus* (n = 1). The armrests of chairs were the sites most frequently contaminated with pathogens. All identified microorganisms in both waiting areas, the median CFUs and the achieved UV-C doses, reported separately for each sampling site, time of sampling and outpatient waiting area, are given in the supplemental material (Additional file 1: Tables S1–S4).

### Effects on *Candida auris*

**Fig. 5:**
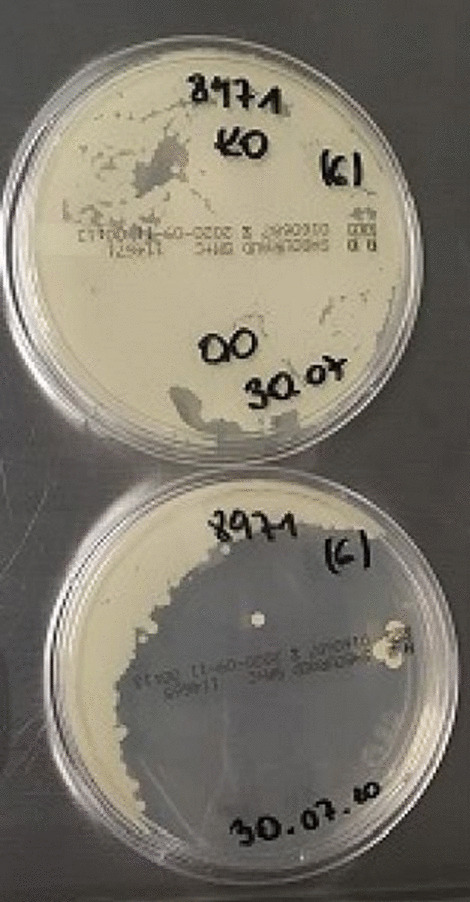
*C. auris* (10^6^ CFUs/ml) on Sabouraud plates without (above) and with (below) exposure to UV-C irradiation following incubation

UV-C irradiation emitted by the robot reduced the growth of all four *C. auris* strains spread at a concentration of 10^6^ CFUs/ml on SDA plates, mimicking the microbial lag phase. However, as shown in Fig. 5, growth of *C. auris* was observed on one fourth of the plate. According to the indicators placed alongside, the measured UV-C dose was 100 mJ/cm^2^ (indicating maximum exposure) except for the area right next to the rim of the plate, demonstrating the shadow effect of the rim.

**Fig. 6:**
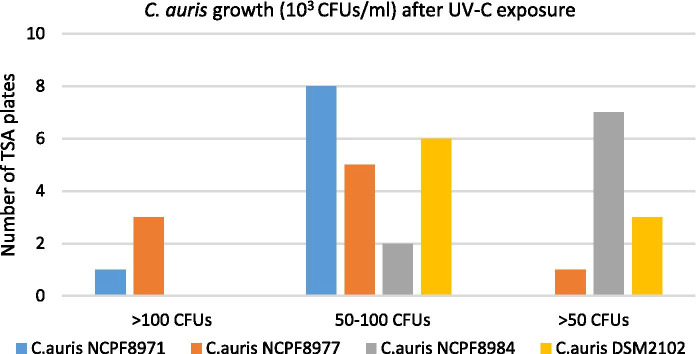
Growth of *C. auris* strains in a stationary phase on TSA contact plates after UV-C exposure

The effect of the UV-C robot on stationary *C. auris* cells at an initial concentration of 10^3^ CFUs/ml was variable and depended on the *C. auris* strain tested (Table 3). The *C. auris* NCPF 8984 strain was the most sensitive of the tested strains. It also showed the most consistent results regarding growth reduction after UV-C exposure compared to control plates. *C. auris* NCPF 8971 consistently showed growth greater than 50 CFUs on each TSA contact plate after UV-C exposure. In terms of the UV-C dose received, the indicators indicated high exposure (75–100 mJ/cm^2^) for all *C. auris* plates (Table 3, Fig. 6).

**Table 3:** Colony counts per TSA plate containing *C. auris* in a stationary phase (10^3^ CFUs/ml) with and without UV-C exposure

	*C. auri*s NCPF 8971	*C. auris* NCPF 8977	*C. auris* NCPF 8984	*C. auris* DSM 21092
Control (without UV-C exposure)	> 100	> 100	> 100	> 100
Day1 (after UV-C)				
1	> 50	35	1	> 50
2	> 50	> 50	28	35
3	> 50	> 50	1	> 50
Day2 (after UV-C)				
1	> 50	> 100	> 50	20
2	> 50	> 100	11	> 50
3	> 100	> 100	10	> 50
Day3 (after UV-C)				
1	> 50	> 50	11	> 50
2	> 50	> 50	0	> 50
3	> 50	> 50	> 50	30

Corresponding UV-C doses received during each disinfection cycle ranged from 75 to 100 mJ/cm^2^

### Use of a UV-C robot for the routine cleaning and/or disinfection process

The UV-C robot required many attempts until it could carry out the UV-C disinfection process independently. Interventions by the operator were necessary due to initial programming imprecisions, furniture that had accidentally been moved during routine clinical operations, detection of movement during an ongoing disinfection cycle or loss of internet connection. Although the area was cordoned off during the disinfection cycles and appropriate warning signs were posted on the access doors, we found it difficult to ensure the total absence of health personnel returning to their nightshift rooms nearby or other individuals who moved in and out the closed area.

In terms of its user-friendliness and simplicity of operation, the device required—in addition to the pre-programming of the area’s maps—further preceding steps to start the disinfection process. The user had to select several items in two different apps on the device’s control panel, which was not self-explanatory.

## Discussion

The contaminated hospital environment is a reservoir for various pathogens and may thus serve as a source of HAIs [30]. Conventional manual cleaning and disinfection processes are not always  sufficient to eliminate the risk posed by contaminated surfaces [3, 8, 9]. Human factors are likely to be a major contributor. Furthermore, during the COVID-19 pandemic, effective standard disinfectants were unavailable in times of crisis [10], indicating the need of new disinfectants or disinfection methods. Most recently, autonomously moving UV-C disinfection devices—UV-C robots—have been developed to overcome these shortcomings.

The present study shows that UV-C irradiation emitted by the robot significantly decreased the residual surface contamination in the waiting areas of two outpatient clinics of a tertiary hospital compared to manual cleaning and disinfection alone. This is in accordance with other studies that have found a significant decrease of the pathogen bioburden in clinical settings by using a robotic UV-C irradiation device [12, 13, 15, 31]. Anderson et al. found that the application of UV-C light significantly reduced the presence of Vancomycin-resistant enterococci (VRE) and *Clostridium difficile* in patient rooms previously occupied by colonized patients compared to baseline (without prior manual cleaning and disinfection) [20]. Similarly, Yang et al. observed a significant reduction of the number of bacteria colonies sampled from different surfaces after UV-C exposure in uncleaned rooms previously occupied by VRE and MRSA carriers [32]. In the present study—despite the fact that not all surfaces achieved full UV-C light exposure—almost all microorganisms still present after manual cleaning and disinfection were eliminated. However the microbial burden on surfaces was low to average on almost all surfaces after manual cleaning and/or disinfection alone. Especially in the ENT outpatient waiting area, manual cleaning and disinfection had been carried out meticulously, resulting in the recovery of only few CFUs. Insufficient UV-C light exposure might have yielded less effective results in other settings, e.g. with higher surface contamination levels.

Using *C. auris* as a surrogate for microorganisms resilient in the environment, the UV-C disinfection robot tested showed varying effectiveness: For *C. auris* that had been incubated for 24 h before exposure to UV-C irradiation, growth inhibition was not effectively achieved compared to *C. auris* without prior incubation (for fully exposed areas). However, this may be due to our different sample preparations, mimicking *C. auris* in a lag versus a stationary growth phase. Furthermore, the formation of shadows from the rim of SDA plates drastically decreased the effectiveness of UV-C light. De Groot et al. investigated the effect of different distances (two or four meters) from the UV-C emitting device as well as different cycle times (5, 10, 20 or 30 min) on *C. auris* in vitro, demonstrating strain-dependent effectiveness. Longer UV-C exposure and less distance improved the effects of UV-C irradiation [21]. The present study evaluated the effects of UV-C irradiation on *C. auris* delivered by a robot in a clinical setting. These results confirm that longer UV-C exposure is necessary to eradicate *C. auris*, especially when inoculums are high and pathogens have already had time to mature. Thus, it is pivotal to validate the effectiveness of UV-C robotic devices separately for each clinical setting. To achieve this, the robot’s settings (distance and duration of exposure) must be carefully calibrated and adapted further until microbiological outcomes are satisfactory. This requires the expertise of a clinical microbiologist and IPC specialist. UV-C sensitive, color-changing indicators measuring the amount of UV-C irradiation received must be used as quality controls. Particularly for surfaces or objects that create shadows and cannot be reached by UV-C irradiation, manual cleaning and disinfection are still needed. In the future, design of hospital areas will have to avoid structures creating shadows if UV-C disinfection is applied. Nevertheless, for surfaces beyond the reach of the cleaners, UV-C robots may be useful. Then, to achieve effective UV-C disinfection the UV-C source must be able to move into three dimensions (tilt, move up and down).

The usability of the UV-C robot in the clinical setting was not as satisfactory as expected from a robotic device. This study aimed to evaluate the UV-C robot in action, particularly its usability when integrated into the standard cleaning and disinfection process. UV-C robots are usually advertised as being simple to use with no additional decontamination needed. However, in our own experience, we repeatedly needed technical assistance to operate the robot. Future technological advances are supposed to overcome these failings. In addition to a somewhat complicated programming of the parameter settings, the robot tested was mostly not autonomous and not able to carry out the room disinfection independently. Apart from technical reasons, this dysfunction was also due to the fact that furniture and other objects had been slightly moved between the initial programming and the robot’s use. Moreover, despite sealing the areas off during the UV-C disinfection cycles, people moved in and out. Yet, this is normal in the hospital reality. Advances in IT, particularly using artificial intelligence together with high-tech cameras may be the clue for solving these problems. Additionally, further technical developments such as making the UV-C light source more flexible and the robot itself smaller will be necessary in addition to adjustments in the clinical environment to minimize the formation of shadows, thus enabling the proper use of this novel technology.

The decision to use a UV-C disinfection robotic device in clinical settings must be made on the basis of the intended application area, the practicability of use and the additional expected benefit. At the present stage of UV-C robot technology, these robots will be preferably used in areas with stationary furniture that can easily be sealed off to avoid people walking in and out during an ongoing disinfection cycle. Practicability means that the UV-C robot will be operated by trained cleaning staff rather than by an engineer or a technician. An additional expected benefit, however, might be achieved in areas with vulnerable patients or in over-busy areas with highly contaminated surfaces including the risk of multidrug-resistant microorganisms, e.g. emergency departments.

There are some limitations to this study. First, showing a reduction in the microbial burden on surfaces is a surrogate outcome. The study design did not allow for an evaluation of the effect of UV-C irradiation in addition to manual cleaning and disinfection on HAI rates compared to manual cleaning and disinfection alone. Next, the classification of the sampling sites into surfaces with a low, average and high microbial burden was made according to our in-house standard, which is used to audit the cleaning efficacy. It refers to the colony count but is not directly associated with patients’ outcomes. Further, the cleaning personnel was not blinded to the intervention, which might have affected their behavior, resulting in more thorough cleaning and not reflecting the quality of cleaning in daily practice. Therefore, our results might underestimate the benefit provided by adding a UV-C component. Next, to determine the effects of the UV-C robotic device on *C. auris*, artificially inoculated plates were used as a surrogate for surface-bound contamination. This might not accurately reflect growth patterns of *C. auris* on real-world surfaces. Moreover, densities occurring on contaminated hospital surfaces may not be as high, resulting in an underestimation of the ability of UV-C light to kill *C. auris*.

## Conclusion

The UV-C disinfection model robot tested in our study was not yet ready for everyday use in hospitals due to several technical shortcomings and difficulty of use as well as likely significant additional expense. We also observed persistence of *C. auris* in a stationary phase, indicating that a standard disinfection cycle might not suffice to inactivate more UV-C resistant pathogens, especially when inoculums are high. While UV-C technologies improve surface decontamination results, they do not simplify current processes and can presently only serve as add-on components to manual cleaning and disinfection carried out by trained and audited cleaning staff. However, there is huge potential in this technology once it is further developed.

## Supplementary Information

**Additional file 1.** The use of a UV-C disinfection robot in the routine clearning process: a field study in an Academic Hospital.** Table S1**. Reductions in Colony-Forming Units in the ENT outpatient area after routine cleaning and/or disinfection, and after the use of the UV-C robot.** Table S2**. Reductions in Colony-Forming Units in the oncology outpatient area after routine cleaning and/or disinfection, and after the use of the UV-C robot.** Table S3**. ENT outpatient area: Environmental microbiome identified during the study period.** Table S4**. Oncology outpatient area: Environmental microbiome identified during the study period.

## Data Availability

The datasets used and/or analyzed during the current study are available from the corresponding author on reasonable request.

## Abbreviations

*C. auris*: *Candida auris*
C&D: Cleaning and disinfection
CFU: Colony-Forming Unit
ENT: Ear, nose and throat
IPC: Infection prevention and control
HAI: Healthcare-associated infection
MRSA: Methicillin-resistant *Staphylococcus aureus*
SDA: Sabouraud Dextrose Agar
SOP: Standard operating procedure
STC&D: Standard terminal room cleaning and disinfection
TSA: Tryptic Soy Agar
UV-C: Ultraviolet-C
VRE: Vancomycin-resistant Enterococcus
