# Supplementary material for: The use of a UV-C disinfection robot in the routine cleaning process: a field study in an Academic hospital
Source: Antimicrob Resist Infect Control. 2021 May 29;10:84. doi: 10.1186/s13756-021-00945-4 (PMC8164075; doi:10.1186/s13756-021-00945-4)
Supplement: Supplementary file 1 — Additional file 1. The use of a UV-C disinfection robot in the routine clearning process: a field study in an Academic Hospital. Table S1. Reductions in Colony-Forming Units in the ENT outpatient area after routine cleaning and/or disinfection, and after the use of the UV-C robot. Table S2. Reductions in Colony-Forming Units in the oncology outpatient area after routine cleaning and/or disinfection, and after the use of the UV-C robot. Table S3. ENT outpatient area: Environmental microbiome identified during the study period. Table S4. Oncology outpatient area: Environmental microbiome identified during the study period. [file 13756_2021_945_MOESM1_ESM.docx]

## Supplement

| **Additional Table 1. Reductions in Colony-Forming Units in the ear, nose and throat outpatient area after routine cleaning and/or disinfection, and after the use of the UV-C robot (including UV-C dose received)** | | | | | | |
| --- | --- | --- | --- | --- | --- | --- |
| **Sampling area** | **Time of Sampling** | **No. of samples** | **Mean CFU (±SD)** | **Median CFU** | **IQR** | **mJ/cm²** |
| Wall | Before C&D | 4 | 0.25 (±0.5) | 0 | 0-0.8 |  |
|  | After C&D | 4 | 0 (±0) | 0 | 0 |  |
|  | After C&D + UV-C | 4 | 0 (±0) | 0 | 0 | 100 |
| Arm rest of chair No. 1 | Before C&D | 4 | 74.0 (±89.4) | 37.0 | 19.0-166.0 |  |
|  | After C&D | 4 | 6.0 (±8.5) | 3.0 | 0-15 |  |
|  | After C&D + UV-C | 4 | 0 (±0) | 0 | 0 | 75 |
| Back of chair | Before C&D | 4 | 0.5 (±0.6) | 0.5 | 0.0-1.0 |  |
|  | After C&D | 4 | 0 (±0) | 0 | 0 |  |
|  | After C&D + UV-C | 4 | 0 (±0) | 0 | 0 | 25 |
| Arm rest of chair No. 2 | Before C&D | 4 | 66.8 (±68.4) | 60.0 | 7.8-132.5 |  |
|  | After C&D | 4 | 2.8 (±3.8) | 1.5 | 0-6.8 |  |
|  | After C&D + UV-C | 4 | 0 (±0) | 0 | 0 | 100 |
| Wooden play element | Before C&D | 4 | 7.8 (±9.7) | 5.5 | 0.0-17.8 |  |
|  | After C&D | 4 | 2 (±2.8) | 1.0 | 0.0-5.0 |  |
|  | After C&D + UV-C | 4 | 0 (±0) | 0 | 0 | 75 |
| Window countertop | Before C&D | 4 | 28.3 (±34.0) | 16.5 | 5.0-63.3 |  |
|  | After C&D | 4 | 2.3 (±2.1) | 2.0 | 0.5-4.3 |  |
|  | After C&D + UV-C | 4 | 0.5 (±0.6) | 0.50 | 0.0-1.0 | 25-50 |
| No.=Number; CFU=Colony Forming Unit; IQR=Interquartile Range; C&D=Cleaning and Disinfection; SD=Standard Deviation | | | | | | |

| **Additional Table 2. Reductions in Colony-Forming Units in the oncology outpatient area after routine cleaning and/or disinfection, and after the use of the UV-C robot (including UV-C dose received)** | | | | | | |
| --- | --- | --- | --- | --- | --- | --- |
| **Sampling site** | **Time of Sampling** | **No. of samples** | **Mean CFU (±SD)** | **Median CFU** | **IQR** | **mJ/cm²** |
| Patient registration area | Before C&D | 5 | 11.0 (±6.3) | 14 | 4.5-16.0 |  |
|  | After C&D | 5 | 7.6 (±1.5) | 8 | 6.0-9.0 |  |
|  | After C&D + UV-C | 5 | 0.8 (±1.3) | 0 | 0-2.0 | 25 |
| Table top next to | Before C&D | 5 | 21.4 (±13) | 25 | 8.0-33.0 |  |
| patient registration | After C&D | 5 | 12.2 (±8.3) | 8 | 6.0-20.5 |  |
|  | After C&D + UV-C | 5 | 1.0 (±2.2) | 0 | 0-2.5 | 75 |
| Table top waiting area | Before C&D | 5 | 20.4 (±11.6) | 23 | 9.5-30.0 |  |
|  | After C&D | 5 | 10.4 (±9.0) | 6 | 4.0-19.0 |  |
|  | After C&D + UV-C | 5 | 0 (±0) | 0 | 0 | 75 |
| Arm rest of chair No. 1 | Before C&D | 5 | 102 (±69.1) | 114 | 35.5-162.5 |  |
|  | After C&D | 5 | 24.6 (±25.6) | 9 | 4.5-52.5 |  |
|  | After C&D + UV-C | 5 | 0 (±0) | 0 | 0 | 75 |
| Arm rest of chair No. 2 | Before C&D | 5 | 108.0 (±71.9) | 118 | 36.0-175.0 |  |
|  | After C&D | 5 | 47.8 (±31.1) | 41 | 26.0-73.0 |  |
|  | After C&D + UV-C | 5 | 0.4 (±0.5) | 0 | 0-1.0 | 100 |
| Window countertop | Before C&D | 5 | 45.2 (±34.1) | 31 | 25.0-72.5 |  |
|  | After C&D | 5 | 12.4 (±19.6) | 5 | 1.0-27.5 |  |
|  | After C&D + UV-C | 5 | 1.4 (±2.0) | 0 | 0-3.5 | 25 |
| Push button of vending | Before C&D | 5 | 6.2 (±8.0) | 3 | 1.0-13.0 |  |
| machine | After C&D | 5 | 2.8 (±4.1) | 1 | 0.5-6.0 |  |
|  | After C&D + UV-C | 5 | 0 (±0) | 0 | 0 | 100 |
| Leaflet dispenser | Before C&D | 5 | 0 (±0) | 0 | 0 |  |
|  | After C&D | 5 | 0.8 (±1.3) | 0 | 0-2.0 |  |
|  | After C&D + UV-C | 5 | 0 (±0) | 0 | 0 | 100 |
| No.=Number; CFU=Colony Forming Unit; IQR=Interquartile Range; C&D=Cleaning and Disinfection; SD=Standard deviation | | | | | | |

| **Additional Table 3. Ear, nose and throat outpatient area: Environmental microbiome identified during the study period** | |
| --- | --- |
| **Sampling site** | **Identified species^1^** |
| Wall | Staphylococcus capitis |
|  |  |
| Arm rest of chair No. 1 | **Staphylococcus saprophyticus** |
|  | Staphylococcus capitis |
|  | Staphylococcus epidermidis |
|  | Staphylococcus haemolyticus |
|  | Staphylococcus hominis |
|  | Kocuria palustris |
|  | Micrococcus luteus |
|  | Micrococcus lylae |
|  | Bacillus cereus |
|  | Bacillus megaterium |
|  | Bacillus simplex |
|  | Bacillus subtilis |
|  | Paenibacillus massiliensis |
|  | Streptomyces violaceoruber |
|  | **Streptococcus pneumoniae** |
|  | Streptococcus mitis |
|  | Moraxella osloensis |
|  |  |
| Back of chair | Staphylococcus epidermidis |
|  | Micrococcus luteus |
|  |  |
| Arm rest of chair No. 2 | **Staphylococcus lugdunensis** |
|  | Staphylococcus cohnii |
|  | Staphylococcus epidermidis |
|  | Staphylococcus haemolyticus |
|  | Staphylococcus hominis |
|  | Kocuria rhizophila |
|  | Micrococcus luteus |
|  | Paracoccus yeei |
|  | Bacillus pumilus |
|  | Pseudomonas oryzihabitans |
|  | Roseomonas mucosa |
|  | **Aerococcus viridans** |
|  | Enterococcus moraviensis |
|  | Enterococcus termitis |
|  |  |
| Wooden play element | Staphylococcus hominis |
|  | Micrococcus luteus |
|  | Kocuria kristinae |
|  | Brevibacterium casei |
|  | Bacillus pumilus |
|  | Bacillus circulans |
|  | Lysinibacillus fusiformis |
|  | Mixta calida |
|  |  |
| Window countertop | **Staphylococcus saprophyticus** |
|  | Staphylococcus hominis |
|  | Staphylococcus haemolyticus |
|  | Staphylococcus epidermidis |
|  | Staphylococcus petrasii |
|  | Micrococcus luteus |
|  | Acinetobacter lwoffii |
|  | Exiguobacterium aurantiacum |
|  | Pantoea dispersa |
|  | Moraxella osloensis |
| ¹typical pathogens in bold | |

| **Additional Table 4. Oncology outpatient area: Environmental microbiome identified during the study period** | |
| --- | --- |
| **Sampling site** | **Identified species**^1^ |
| Patient registration area | Staphylococcus capitis |
|  | Staphylococcus epidermidis |
|  | Staphylococcus haemolyticus |
|  | Staphylococcus hominis |
|  | Staphylococcus simulans |
|  | Staphylococcus warneri |
|  | Brevibacterium luteolum |
|  | Corynebacterium tuberculostearicum |
|  | Dermabacter hominis |
|  | Kokuria marina |
|  | Micrococcus luteus |
|  | Moraxella osloensis |
|  |  |
| Table top next to patient registration | Staphylococcus capitis |
|  | Staphylococcus epidermidis |
|  | Staphylococcus haemolyticus |
|  | Staphylococcus hominis |
|  | Staphylococcus petrasii |
|  | Kocuria kristinae |
|  | Micrococcus luteus |
|  | Acinetobacter radioresistens |
|  | Exiguobacterium aurantiacum |
|  | Moraxella osloensis |
|  |  |
| Table top waiting area | **Staphylococcus saprophyticus** |
|  | Staphylococcus epidermidis |
|  | Staphylococcus haemolyticus |
|  | Staphylococcus hominis |
|  | Staphylococcus petrasii |
|  | Arthrobacter gandavensis |
|  | Acinetobacter parvus |
|  | Micrococcus luteus |
|  | Micrococcus lylae |
|  | Bacillus pumilus |
|  | Bacillus megaterium |
|  | **Acinetobacter baumanii** |
|  | Moraxella osloensis |
|  |  |
| Arm rest of chair No. 1 | **Staphylococcus aureus** |
|  | Staphylococcus capitis |
|  | Staphylococcus condimenti |
|  | Staphylococcus epidermidis |
|  | Staphylococcus hominis |
|  | Staphylococcus warneri |
|  | Corynebacterium mucifaciens |
|  | Dermacoccus nishinomiyaensis |
|  | Kokuria rhizophila |
|  | Micrococcus luteus |
|  | Micrococcus lylae |
|  | Bacillus flexus |
|  | Bacillus licheniformis |
|  | Fictibacillus arsenicus |
|  | Pseudomonas stutzeri |
|  | Moraxella osloensis |
|  | Rothia mucilaginosa |
|  |  |
| Arm rest of chair No. 2 | **Staphylococcus saprophyticus** |
|  | Staphylococcus capitis |
|  | Staphylococcus cohnii |
|  | Staphylococcus epidermidis |
|  | Staphylococcus haemolyticus |
|  | Staphylococcus hominis |
|  | Staphylococcus petrasii |
|  | Staphylocuccus pettenkoferi |
|  | Staphylococcus warneri |
|  | Kokuria rhizophila |
|  | Micrococcus luteus |
|  | Micrococcus lylae |
|  | Bacillus cereus |
|  | Bacillus simplex |
|  | Bacillus pumilus |
|  | Brevibacillus borstelensis |
|  | Lysinibacillus fusiformis |
|  | Mixta calida |
|  | Pantoea eucrina |
|  | Stenotrophomonas acidaminiphila |
|  | Moraxella osloensis |
|  | Moraxella sp. |
|  | **Enterococcus casseliflavus** |
|  |  |
| Push button of vending machine | Staphylococcus capitis |
|  | Staphylococcus epidermidis |
|  | Staphylococcus haemolyticus |
|  | Staphylococcus hominis |
|  | Corynebacterium mucifaciens |
|  | Corynebacterium propinquum |
|  | Paenibacillus glucanolyticus |
|  | Moraxella osloensis |
|  | **Acinetobacter baumanii** |
|  |  |
| Leaflet dispenser | Staphylococcus hominis |
|  | Micrococcus luteus |
| ¹typical pathogens in bold | |
